# Supplementary material for: Data from Entomological Collections of Aedes (Diptera: Culicidae) in a post-epidemic area of Chikungunya, City of Kinshasa, Democratic Republic of Congo
Source: GigaByte. 2023 Nov 8;2023:gigabyte96. doi: 10.46471/gigabyte.96 (PMC10644237; doi:10.46471/gigabyte.96)
Supplement: Translation

# Translation: Données des collectes entomologiques des Aedes (Diptera : Culicidae) dans une zone post-épidémique de Chikungunya, Ville de Kinshasa, République Démocratique du Congo

**Authors:** Victoire Nsabatien, Josue Zanga, Fiacre Agossa, Nono Mvuama, Maxwell Bamba, Osée Mansiangi, Leon Mbashi, Vanessa Mvudi, Glodie Diza, Dorcas Kantin, Narcisse Basosila, Hyacinthe Lukoki, Arsene Bokulu, Christelle Bosulu, Erick Bukaka, Jonas Nagahuedi, Jean Claude Palata, Emery Metelo

**Affiliations:** ^1^ Laboratoire d’écologie animale appliquée, Département des sciences de la vie, Faculté des sciences et technologies, Université de Kinshasa, RD Congo; ^2^ Laboratoire de Bioécologie et de lutte antivectorielle, Département de santé et environnement, École de Santé Publique de Kinshasa, RD Congo; ^3^ Unité d’entomologie, Département de parasitologie, Institut national de recherche biomédicale, Kinshasa, RD Congo; ^4^ PMI VectorLink Project, Abt Associates, 6130 Executive Blvd, Rockville, MD 20852, USA; ^5^ Laboratoire d’ethnologie et de photochimie médicale, Département des sciences de la vie, Faculté des sciences et technologies, Université de Kinshasa, RD Congo; ^6^ Programme national de lutte contre le paludisme, RD Congo; ^7^ Laboratoire de botanique, de systématique et d’écologie végétale, Département des sciences de la vie, Faculté des sciences et technologies, Université de Kinshasa, RD Congo; ^8^ Unité d’entomologie, Département des sciences de la vie, Faculté des sciences et technologies, Université de Kinshasa, RD Congo

## Abstract

Les épidémies d’arbovirus (chikungunya, dengue, fièvre du Nil occidental, fièvre jaune et zika) constituent une menace croissante dans les régions africaines où *Aedes* (Stegomyia) *aegypti* (Linnaeus, 1762) et *Aedes albopictus* (Skuse, 1895) sont présents. L’absence d’échantillonnage complet de ces deux vecteurs limite notre compréhension sur la dynamique de propagation de ces derniers dans les zones à risque d’émergence d’arbovirus. Ici, nous avons collecté 6 943 observations (collecte larvaire et capture sur appât humain) de *Ae. aegypti* et de *Ae. albopictus* entre 2020 et 2022. L’étude a été réalisée dans la Vallée de la Funa, une zone post-épidémique de la ville de Kinshasa, en République démocratique du Congo. Nos résultats fournissent des informations importantes pour de futures études fondamentales et avancées sur l’écologie et la phénologie de ces vecteurs, ainsi que sur la dynamique de ces vecteurs après une période post-épidémique. Les données de cette étude sont publiées dans le domaine public en tant que Darwin Core Archive dans le Global Biodiversity Information Facility.

## Description des données

### Cadre et contexte

Les épidémies d’arbovirus, en particulier ceux conduisant à des maladies telles que le chikungunya, la dingue, constituent une menace croissante dans les zones africaines où *Aedes aegypti* et *Aedes albopictus* sont présents [1.–4.]. L’absence d’échantillonnage complet de ces deux vecteurs limite notre compréhension sur la dynamique de propagation de ces derniers dans les zones à risque d’émergence d’arbovirus. Des études comme la nôtre ont donc contribuées à la compréhension de la dynamique de ces vecteurs. Nous décrivons ici, pour la première fois, la distribution géographique entre 2020 et 2022 de deux vecteurs d’arbovirus (*Ae. aegypti* et *Ae. albopictus*) dans une zone post-épidémique de chikungunya de la ville province de Kinshasa, en République démocratique du Congo.

## Méthodes

### Couverture spatiale générale

Cette étude a été réalisée dans la Vallée de la Funa (Figure 1), une galerie forestière située dans la zone de collines de la commune de Mont Ngafula. Elle se situe dans un îlot de forêt secondaire dominé par des arbres telles que *Millettia laurentii* et *Pentaclethra eetveldeana*.

**Figure 1.** Carte de la vallée de la Funa, montrant les points de collecte entomologique (nord, sud, est et ouest).

 La galerie forestière est située entre la latitude 4°25*′*06.17*′′*S et la longitude 15°18*′*05.77*′′*E, et couvre environ 45 hectares, et la pluviométrie moyenne annuelle de la zone est d’environ 1 095 mm. De façon générale à Kinshasa, les précipitations sont abondantes et inégalement réparties tout au long de l’année. Le volume de précipitations le plus élevé se produit en novembre, avec une moyenne de 268,1 mm, et le plus faible en juillet, avec une moyenne de 0,7 mm. L’humidité relative annuelle moyenne est de 79%.

La cartographie des points d’échantillonnage de cette étude a été réalisée à l’aide du logiciel ArcGIS version 10.8 (RRID:SCR_011081) [5.], à partir des coordonnées géographiques (longitude est et latitude sud) enregistrées à chaque point de collecte entomologique à l’aide d’un GPS Garmin Etrex GPmap 64s. Les couches vectorielles utilisées sur la carte ont été dérivées de la base de données du Référentiel Géographique Commun de la République Démocratique du Congo.

### Collecte de moustiques

La description taxonomique générale pour ce travail est de la famille des Culicidae, spécifiquement *Ae. aegypti* (communément appelé moustique de la fièvre jaune ; NCBI:txid7159) et *Ae. albopictus* (communément appelé moustique tigre d’Asie ou moustique tigre en français ; NCBI:txid7160).

Deux techniques d’échantillonnage ont été utilisées pour collecter les stades immatures et adultes d’Aedes spp. entre le 5 janvier 2020 et le 30 décembre 2022. Quelle que soit les techniques d’échantillonnage utilisées, l’identification morphologique des moustiques adultes a été réalisée à l’aide des clés taxonomiques [6.].

### Collecte larvaire

Au cours de la période d’étude 2020–2022, les larves ont été collectées une fois par an en octobre, mois qui annonce le retour des fortes pluies après une saison sèche qui se tend de mi-mai à mi-août. Les stades immatures des Aedes spp. ont été collectés dans divers sites de reproduction (pots abandonnés, pneus, boîtes de conserve et autres récipients) à l’aide de la technique de dipping. Cette technique consiste à prélever de l’eau dans les gîtes larvaires à l’aide d’une louche ou d’un petit récipient et à rechercher les larves de Culicidae de façon générale, en particulier celles des Aedes spp (cas de cette étude). Ces larves ont été collectées et stockées dans des bocaux contenant l’eau de leurs sites de reproduction respectifs, puis transportées à l’insectarium du Laboratoire de Bioécologie et de Lutte Antivectorielle (ou BIOLAV), où elles ont été élevées jusqu’à l’âge adulte dans des conditions d’insectarium (température : 28 ± 1 °C ; humidité relative : 70–80% ; photopériode 14 h : 10 h lumière : obscurité). Dans l’insectarium, les larves ont été placées dans des bacs d’élevage en fonction du type d’habitat larvaire qu’elles ont été trouvées sur le terrain ; le pipetage des pupes et l’émergence des adultes ont suivi les mêmes procédures de classification. Cinq jours après l’émergence, les moustiques adultes ont été aspirés dans des cages, placés dans des bocaux et refroidis à −23 °C pour éviter qu’ils ne s’échappent pendant l’identification. Les données d’identification ont été encodées dans une feuille Excel et les échantillons de moustiques ont été conservés à −23 °C en vue d’une éventuelle analyse ultérieure.

### Capture sur appât humain (CAH)

Après approbation éthique et consentement, les collectes des Aedes à la recherche d’hôtes ont été effectuées au cours de quatre échantillonnages par an, pendant trois ans, et réparties comme suit : janvier «courte saison des pluies accompagnée d’une courte saison sèche» ; juin « saison sèche» ; août «début de la saison des pluies» ; octobre « saison des pluies». Au cours de chaque campagne d’échantillonnage, les collecteurs ont été supervisés à l’intérieur et à l’extérieur de chaque maison afin d’assurer le bon déroulement des activités de capture. Les superviseurs ont répondu aux préoccupations des collecteurs et ont effectué des visites inopinées à chaque station de capture pour s’assurer que la qualité du travail était conforme aux instructions.

La capture sur appât humain (CAH) a été utilisée pour collecter les adultes des Aedes spp. pendant la journée [7.]. Sur le site de l’étude, les collectes de moustiques ont été effectuées à l’intérieur et à l’extérieur des ménages, un premier groupe de collecteurs travaillant de 7h00 à 12h00 et remplacé par un second groupe de 12h00 à 19h00. À chaque point de collecte, un volontaire pieds nus a servi d’appât et a collecté des moustiques à l’aide de tubes à hémolyse. Les échantillons de moustiques ont ensuite été transportés dans la salle d’identification morphologique du laboratoire de Bioécologie et de Lutte antivectorielle pour l’identification morphologique.

### Description du contrôle qualité

Les moustiques ont été identifiés à l’aide de clés disponibles dans la littérature [6.] par un entomologiste expérimenté dans l’identification des Aedes d’Afrique centrale. Une fois numérisées, les données ont été validées à l’aide de l’outil de validation IPT (Integrated Publishing Toolkit) disponible auprès du GBIF (Global Biodiversity Information Facility) [8.].

## Résultats

Au total, 6 943 observations (résumées dans le Tableau 1) ont été rapportées à l’aide des méthodes de collecte larvaire et capture sur appât humain (CAH) entre 2020 et 2022. La méthode de collecte CAH a permis de collecter 4 638 moustiques adultes, tandis que l’échantillonnage des larves a produit 2 305 individus. *Ae. albopictus* été l’espèce la plus abondante dans la région étudiée, avec 6 358 individus, alors que nous avons enregistré 578 individus de *Ae. aegypti*. Seuls sept individus n’ont pu être identifiés au niveau de l’espèce.

**Figure 2.** Carte interactive des occurrences géoréférencées hébergées par le GBIF [8.]. https://www.gbif.org/dataset/438cb683-7d8e-4677-bc30-013b675973fc

**Tableau 1:** Synthèse des résultats obtenus avec deux techniques d’échantillonnage entomologique après trois années d’étude dans une zone post-épidémique de Chikungunya.

### Possibilité de réutilisation

Les résultats de cette étude fourniront des informations importantes pour de futures études fondamentales et avancées sur l’écologie et la phénologie de ces vecteurs, ainsi que sur la dynamique des vecteurs après une période épidémique. Etant donné que ces vecteurs ont été responsables d’une charge de morbidité considérable au cours des différentes périodes épidémiques de chikungunya en République Démocratique du Congo, les résultats de ces enquêtes entomologiques peuvent aider les décideurs nationaux à cibler les interventions appropriées et à allouer des ressources aux programmes nationaux de lutte antivectorielle. Nos résultats peuvent également aider à expliquer pourquoi les interventions existantes ne fonctionnent pas comme prévu.

## Data Availability

Les données présentées dans cet article ont été publiées par l’IPT de l’Université de Kinshasa et sont disponibles via le GBIF sous le couvert d’une dérogation CC0 [8.].

## Description de la base de données

**Nom de l’objet :** Données des collections entomologiques d’Aedes (Diptera : Culicidae) dans une zone post-épidémique de Chikungunya, Ville de Kinshasa, République Démocratique du Congo (en anglais seulement)

**Codage des caractères :** UTF-8

**Nom du format :** Darwin Core Archive format

**Version du format :** 1.0

**Distribution:** https://cloud.gbif.org/africa/archive.do?r=aedes_drc

**Date de publication des données :** 2023-06-30

**Langue:** English

**Licences d’utilisation :** Public Domain (CC0 1.0)

**Langue des métadonnées :** English

**Date de création des métadonnées :** 2023-06-30

**Niveau hiérarchique :** Dataset

## Note de l’éditeur

Cet article fait partie d’une série d’articles sur la diffusion des données qui travaillent avec le GBIF et sont soutenus par le TDR, le Programme spécial de recherche et de formation sur les maladies tropicales hébergé par l’Organisation mondiale de la santé [9.].

## Abbreviations

BIOLAV, Laboratoire de Bioécologie et de Lutte antivectorielle; GBIF, Global Biodiversity Information Facility; CAH, capture sur appât ; IPT, Integrated Publishing Toolkit.

## Déclarations

### Approbation éthique et consentement à la participation

L’approbation éthique de cette étude a été obtenue auprès du Comité d’éthique de l’Ecole de santé publique de Kinshasa (Université de Kinshasa), Ref : ESP/CE/91/2020. Des réunions ont été organisées avec les chefs de famille et les agents de capture, au cours desquelles l’objectif et les procédures de l’étude ont été expliqués. La permission a été demandée aux chefs de ménage pour inspecter les habitats larvaires des moustiques et prélever des larves et pour capturer les moustiques adultes dans leurs ménages respectifs.

### Informations sur les auteurs

JZ, NM, VN, VM, OM, MB, LM, GD et DK sont impliqués dans des études de surveillance et contrôle des vecteurs, ainsi que dans des études sur la durabilité des moustiquaires imprégnées d’insecticide au niveau national, et sont tous des chercheurs du laboratoire de Bioécologie et lutte antivectorielle de l’École de santé publique de Kinshasa (Université de Kinshasa). EM, FA, HL, AB et CB mènent des activités de cartographie des vecteurs et de surveillance de la résistance au niveau national, et sont tous des chercheurs dans l’unité d’entomologie de l’Institut national de recherche biomédicale. JN et EB mènent des études sur le contrôle des larves et la cartographie des sites de reproduction des Culicidae dans la ville de Kinshasa, et sont des chercheurs dans l’unité d’entomologie de l’Université de Kinshasa. VN et JP mènent des études sur l’écologie des populations d’arthropodes d’intérêt médical et sont tous des chercheurs au laboratoire d’écologie animale appliquée de l’université de Kinshasa. NB mène des études sur la cartographie des anophèles vecteurs du paludisme au niveau national et le suivi des espèces invasives, et est le point focal du Programme national de lutte contre le paludisme. FA est également chef de projet VectorLink RD.Congo.

### Financement

L’étude a été autofinancée par le Laboratoire de bioécologie et de lutte antivectorielle de l’École de santé publique de Kinshasa.

## References

[1] KamgangB, NchoutpouenE, SimardF Notes on the blood-feeding behavior of *Aedes albopictus* (Diptera: Culicidae) in Cameroon. Parasites Vectors, 2012; 5: 57, doi:10.1186/1756-3305-5-57.22433236PMC3342132

[2] ZahouliJ, KoudouB, MüllerP Urbanization is a main driver for the larval ecology of Aedes mosquitoes in arbovirus-endemic settings in south-eastern Côte d’Ivoire. PLoS Negl. Trop. Dis., 2017; 11(7): e0005751. doi:10.1371/journal.pntd.0005751.28704434PMC5526600

[3] FritzM, Taty TatyR, PortellaC Re-emergence of chikungunya in the Republic of the Congo in 2019 associated with a possible vector-host switch. Int. J. Infect. Dis., 2019; 84: 99–101. doi:10.1016/j.ijid.2019.05.013.31096054

[4] ProesmansS, KatshongoF, MilambuJ Dengue and chikungunya among outpatients with acute undifferentiated fever in Kinshasa, Democratic Republic of Congo: a crosssectional study. PLoS Negl. Trop. Dis., 2019; 13(9): e0007047. doi:10.1371/journal.pntd.0007047.31487279PMC6748445

[5] ESRI. ArcGIS Desktop 10.8.x, 2021; https://desktop.arcgis.com/en/arcmap/latest/get-started/setup/arcgis-desktop-system-requirements.htm.

[6] HuangY, WardR. A pictorial key for the identification of the mosquitoes associated with yellow fever in Africa. Mosq. Syst., 1981; 13(2): 138–149.

[7] RussellTLL, StauntonK, BurkotTR. Standard Operating Procedure for performing Human Landing Catch (HLC). protocols.io. 2022; 10.17504/protocols.io.j8nlkkypwl5r/v1.

[8] NsabatienV, ZangaJ, AgossaF Data from Entomological Collections of Aedes (Diptera: Culicidae) in a post-epidemic area of Chikungunya, City of Kinshasa, Democratic Republic of Congo. Version 1.1. University of Kinshasa. 2023; Occurrence dataset 10.15468/q53h26. Accessed via GBIF.org on 2023-10-16.PMC1064423738023066

[9] Vectors of Human Disease Series. *GigaByte*. 2022; 10.46471/GIGABYTE_SERIES_0002.
